# Lower Limb Lymphedema Patients Can Still Benefit from Supermicrosurgical Lymphaticovenous Anastomosis (LVA) after Vascularized Lymph Node Flap Transfer (VLNT) as Delayed Lymphatic Reconstruction—A Retrospective Cohort Study

**DOI:** 10.3390/jcm10143121

**Published:** 2021-07-15

**Authors:** Johnson Chia-Shen Yang, Shao-Chun Wu, Akitatsu Hayashi, Wei-Che Lin, Gong-Kai Huang, Pei-Yu Tsai, Peng-Chen Chien, Ching-Hua Hsieh

**Affiliations:** 1 Division of Plastic and Reconstructive Surgery, Department of Surgery, Kaohsiung Chang Gung Memorial Hospital, Kaohsiung 833253, Taiwan; mermaid85@cgmh.org.tw (P.-Y.T.); venu_chien@hotmail.com (P.-C.C.); m93chinghua@gmail.com (C.-H.H.); 2 Graduate Institute of Clinical Medical Sciences, College of Medicine, Chang Gung University, Taoyuan 33302, Taiwan; 3 Department of Anesthesiology, Kaohsiung Chang Gung Memorial Hospital, Kaohsiung 833253, Taiwan; shaochunwu@gmail.com; 4 College of Medicine, Chang Gung University, Taoyuan 33302, Taiwan; linwc137@gmail.com (W.-C.L.); b9002061@cgmh.org.tw (G.-K.H.); 5 Department of Lymphedema Center, Kameda General Hospital, Chiba 296-0041, Japan; promise6me5now@gmail.com; 6 Department of Diagnostic Radiology, Kaohsiung Chang Gung Memorial Hospital, Kaohsiung 833253, Taiwan; 7 Department of Pathology, Kaohsiung Chang Gung Memorial Hospital, Kaohsiung 833253, Taiwan

**Keywords:** lymphedema, lymphaticovenous anastomosis, lymphovenous bypass, supermicrosurgery, vascularized lymph node transfer

## Abstract

Background: For lymphedema patients who received a vascularized lymph node flap transfer (VLNT) as their primary treatment, what are the treatment options when they seek further improvement? With recent publications supporting the use of lymphaticovenous anastomosis (LVA) for treating severe lymphedema, we examined whether LVA could benefit post-VLNT patients seeking further improvement. Methods: This retrospective cohort study enrolled eight lymphedema patients with nine lymphedematous limbs (one patient suffered from bilateral lower limb lymphedema) who had received VLNT as their primary surgery. Patients with previous LVA, liposuction, excisional therapy, or incomplete data were excluded. LVA was performed on nine lower lymphedematous limbs. Demographic data and intraoperative findings were recorded. Preoperative and postoperative limb volumes were measured with magnetic resonance volumetry. The primary outcome was the limb volume measured 6 months post-LVA. Results: The median duration of lymphedema before LVA was 10.5 (4.9–15.3) years. The median waiting time between VLNT and LVA was 41.4 (22.3–97.9) months. The median volume gained in the lymphedematous limb was 3836 (2505–4584) milliliters (mL). The median post-LVA follow-up period was 18 (6–30) months. Significant 6-month and 1-year post-LVA percentage volume reductions were found compared to pre-LVA volume (both *p* < 0.001). Conclusion: Based on the results from this study, the authors recommend the use of LVA as a secondary procedure for post-VLNT patients seeking further improvement.

## 1. Introduction

**Figure 1:**
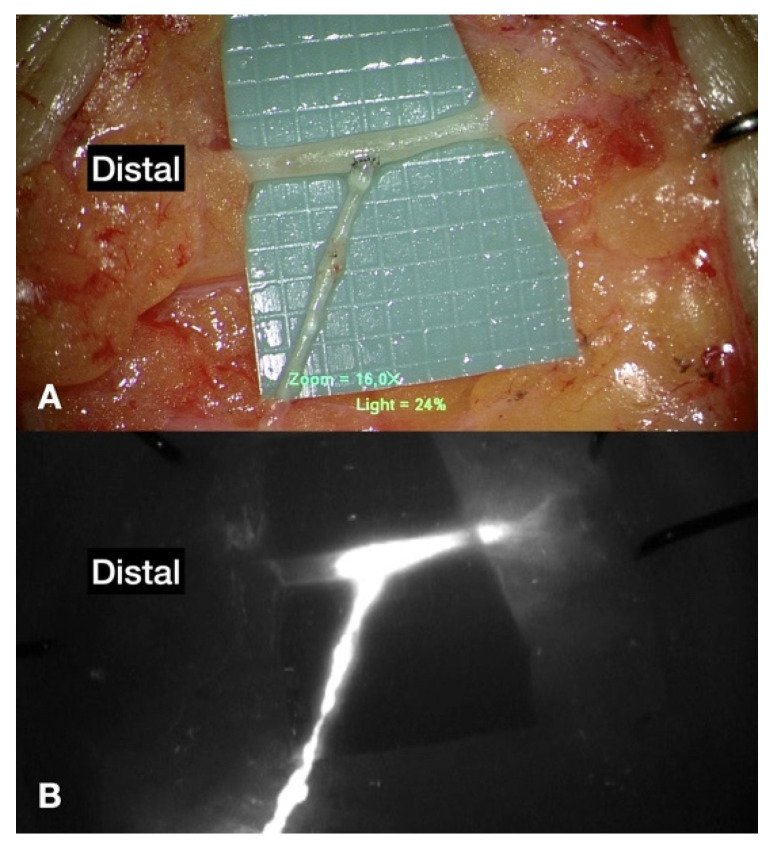
(**A**) Lymphaticovenous anastomosis (LVA) showing a lymphaticovenous end-to-side anastomosis (LVESA). (**B**) Indocyanine green (ICG) lymphography showing ICG entering the recipient’s vein from the lymphatic vessel after anastomosis. Please note that each square on the green background is 1 × 1 mm^2^.

Lymphedema is a chronic, debilitating disease that affects as many as 1 in 30 people worldwide [1]. Currently, the surgical treatments for lymphedema include physiological restoration procedures such as supermicrosurgical lymphaticovenous anastomosis (LVA) and vascularized lymph node flap transfer (VLNT). LVA is a bypass procedure where lymphedema can be improved by channeling the stagnant lymph via the lymphatic vessels (LVs) into the recipient veins (Figure 1). Other methods include liposuction [2] and excisional therapies, such as the Charles procedure [3,4]. Based on previous consensus, VLNT was recommended for moderate-to-severe cases, whereas LVA is reserved exclusively for mild lymphedema [2,5]. However, emerging evidence supports the use of LVA on severe lymphedema patients [6,7,8].

To optimize postoperative outcomes for moderate-to-severe lymphedema for VLNT, multiple modalities, such as one- and two-stage approaches with the use of VLNT, were proposed. One-stage approaches include the concurrent use of triple inset VLNT [9], VLNT and LVA [10,11]; VLNT and the Charles procedure [12,13]; and VLNT with the establishment of a lymph node (LN) efferent lymphatic vessel outflow [14,15]. For two-stage approaches, liposuction before VLNT [16,17] or VLNT after liposuction, with a 1–3-month interval, have been advised. However, despite these efforts, few mixed post-VLNT results were reported [18,19]. We encountered a few patients who received VLNT as their primary lymphedema treatment but with minimal improvement, leading them to demand better outcomes. In this study, we examined whether these post-VLNT patients could benefit from LVA.

## 2. Materials and Methods

This retrospective longitudinal cohort study was approved by the internal review board of our institution (Approval number: 202001420B0). From November 2014 to January 2019, a total of 131 lower limb lymphedema patients were treated in our hospital. Patients who had received VLNT as their primary treatment were enrolled in this study. Patients who had previous LVA, liposuction, Charles procedure, or incomplete data were excluded. The severity of lymphedema was classified based on the International Society of Lymphology (ISL) staging system (mild, Stages 0–I; moderate-to-severe, Stages II–III). All patients underwent supermicrosurgical LVA, performed by a senior surgeon, with 11-0 nylon sutures (Ethilon, Ethicon, Atlantic City, NJ, USA) using a high-power surgical microscope (Pentero 900, Carl Zeiss AG, Oberkochen, Germany).

Demographic data such as sex, age, etiology of lymphedema, ISL staging, body mass index, presence of diabetes mellitus (DM) and hypertension (HTN), the side of the affected lower limb, adjuvant chemotherapy and radiotherapy, duration of lymphedema before LVA, cellulitis episode, VLNT donor sites, time gap between VLNT and LVA, and the volume gained in the limb with lymphedema were recorded. The volume gained in the lymphedematous limb was measured by magnetic resonance (MR) volumetry and calculated by subtracting the volume of the contralateral normal limb from the preoperative lymphedematous limb. 

Intraoperative findings under a surgical microscope included the total LVs found, incisions per patient, LVs found per patient, diameter of LVs, LVA performed per patient (either end-to-end or end-to-side configuration), number and diameter of indocyanine green (ICG)-positive/flow-positive LVs, lymphosclerosis classification (s0, s1, s2, and s3) [20], total number and median diameter of the recipient veins, and recipient veins per patient. As for postoperative care, all patients were asked to wear custom-fabricated compression stockings 1 week after the LVA. The compression garment was recommended to be worn at least during daytime activity. Periodical revisions of compression stockings were recommended. Postoperative MR volumetry was performed 6 months after LVA as a primary outcome. Written consent was obtained from all the patients for the use of their preoperative and postoperative photos.

### 2.1. Operative Technique

Immediately before operation, 0.1 mL of ICG was injected intradermally into the first and third toe web spaces, and the medial and lateral malleolus. A handheld near-infrared imaging device (Fluobeam, FluoOptic, Grenoble, France) was used to detect the dermal backflow (DB) pattern immediately after injection. The ICG-enhanced LVs with linear patterns were traced and marked with a medical grade marking pen and were used as the basis for the incision placement. For patients with a diffuse DB pattern and no linear pattern in sight, incisions were made along the anatomical location of the great saphenous vein. Blind dissection was performed with the aid of a microscope-integrated near-infrared imaging device. The selection of the anastomotic configuration for LVA was based on the size and comparative discrepancy between the LVs and recipient veins as previously published [21]. Three incisions were made for each patient. Additional incisions were made if no suitable LV or vein could be identified for the LVA. The incision was 2–3 cm in length, which could be extended when necessary, as described in our previous publication [6].

### 2.2. Magnetic Resonance Volumetry (Structural Magnetic Resonance Image Acquisition and Volume Calculation) for Lower Limbs

**Figure 2:**
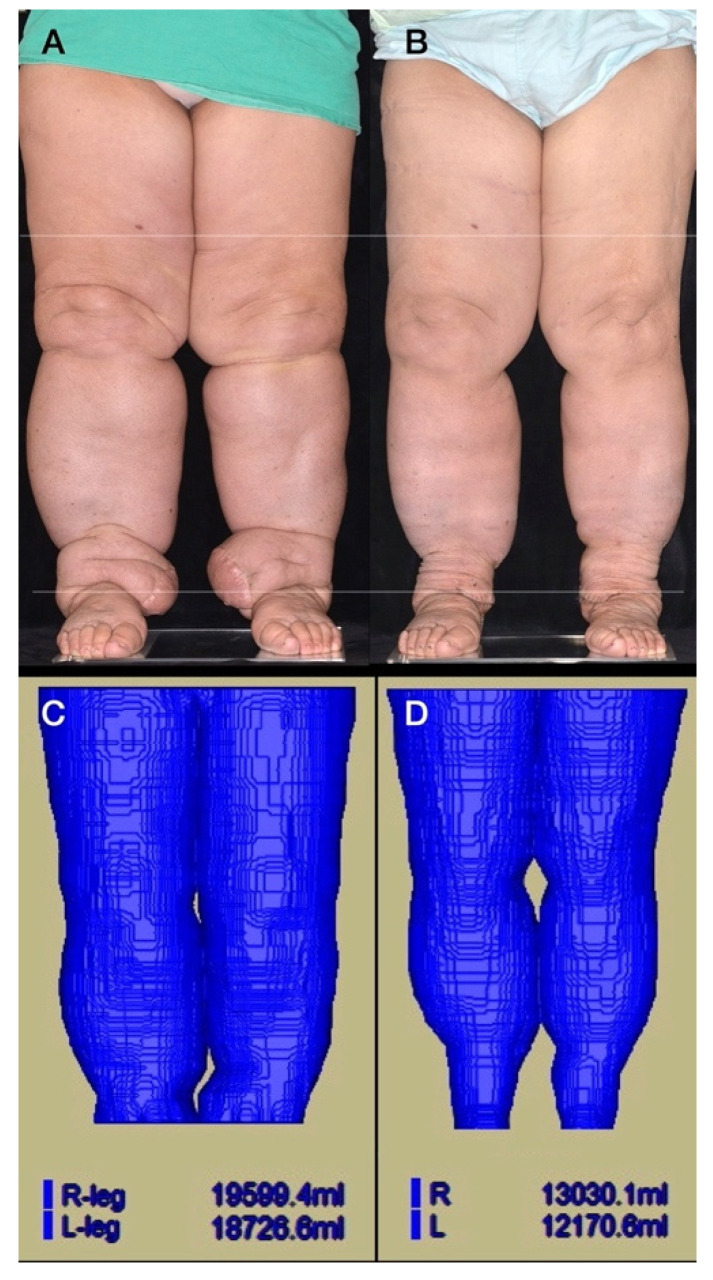
(**A**) A 71-year-old woman with a BMI of 36.1 kg/m^2^ underwent wide excision and lymph node dissection for cervical cancer 25 years ago. Postoperative chemoradiotherapy was performed. Bilateral lower limb lymphedema was noted 11 years ago with recurrent cellulitis (12 times). Submental VLNTs were performed above her bilateral medial malleolus 6 years ago. Due to progressive swelling in her lower limbs after VLNT, LVAs were performed 3 years ago at our hospital. LVA was performed on her right lower limb, which was followed by her left lower limb 1 month later. A total of eight and nine LVAs were performed on her right and left lower limbs, respectively. (**B**) Two-year and 3-month follow-up is shown, after lymph node flap debulk procedure. (**C**) Pre-LVA MR volumetry. Right lower limb, 19,599 mL; left lower limb, 18,727 mL; the average contralateral normal limb volume (5528 mL) of three female lymphedema patients of similar age (68 years old) and height (150 cm) was used as a reference value for calculation. The volume gained due to lymphedema was +14,071 mL (19,599–5528 mL) in the right lower limb; and +13,199 mL (18,727–5528 mL) in the left lower limb. (**D**) Post-LVA MR volumetry at the 2-year and 3-month follow-up: right lower limb, 13,030 mL ((−6569 mL); left lower limb, 12,171 mL (−6556 mL). Post-LVA volume reduction in percentage in her right lower limb = 46.7% (6569 mL × 100/14,071 mL); left lower limb = 49.7% (6556 mL × 100/13,199 mL).

Before the MR examination, the subject was placed in a sitting position for half an hour. The MR examination was performed with the subject in the supine position. All patients underwent MR imaging using a single 3.0 T Siemens MAGNETOM Skyra scanner with two 18-channel body matrix coils (Siemens Healthcare, Erlangen, Germany). For the lower limbs, bilateral lower leg anatomical T1-weighted images were acquired using a coronal three-dimensional sampling perfection with application optimized contrasts using different flip angle evolutions (SPACE) (repeat time/echo time = 500–622/11 ms; field of view = 40 cm; matrix size = 320 × 320; voxel size = 1.3 × 1.3 × 3.0 mm^3^; 60 contiguous slices without an inter-slice gap). An experienced radiologist reviewed all MR scans to exclude any organic disorders other than lymphedema. The volume of the extremities was calculated using a commercialized AZE VirtualPlace software (Aze Ltd., Tokyo, Japan). By using the free-hand mode and auto-threshold function, the volume on each layer image was measured and the total volume of the extremity was automatically calculated. To avoid contamination with pelvic soft tissue in the volumetric analysis, the upper margin was set 20 cm above the knee joint surface of the distal femoral condyle, and the lower margin was set at the ankle articular surface of the inferior tibia. The data were saved to verify that the new reference levels in follow-up studies were in close agreement with the original reference level. All the examinations were performed jointly by the same two radiographers to confirm a match with the original reference level, as described in our previous publication [6] (Figure 2).

### 2.3. Statistical Analysis

The normal distribution of the continuous numeric data was tested using the Kolmogorov–Smirnov normality test. A two-sample independent *t*-test was used to compare normal distributed variables which are expressed as means ± standard deviations. The Mann–Whitney U-test or Kruskal–Wallis test was used to compare data without normal distribution and expressed as a median (25–75%). Chi-square or Fisher’s exact test was used for categorical parameters, including sex, etiology, NECST classification, comorbidities, and previous cancer treatment. SPSS (version 22.0, IBM Corp., Armonk, NY, USA) was used in this study. A *p* < 0.05 was considered statistically significant.

## 3. Results

### 3.1. Demographic Data

A total of eight patients (seven females and one male; median age, 69.5 (58.8–71.3) years) with a previous VLNT were enrolled. Four VLNT patients with incomplete MR volumetry data and one VLNT patient with incomplete LVA data were excluded. All seven female patients had suffered from gynecologic cancers (cervical cancer, endometrial cancer, and ovarian cancer). The male patient was a case of thigh sarcoma. The severity of lymphedema included one mild and seven moderate-to-severe lymphedema patients. The median BMI was 26.5 (22.3–33.6) kg/m^2^. Diabetic mellitus and hypertension were found in two (25%) and three (37.5%) patients, respectively. The affected limbs included three left (37.5%), four right (50%), and one bilateral (12.5%) lower limb. Adjuvant chemotherapy and radiotherapy were performed on three (37.5%) and four (50%) patients, respectively. The median duration of lymphedema before LVA was 10.5 (4.9–15.3) years. The median cellulitis episode before and after LVA was two (1–12) and zero (0–1.5) times, respectively (*p* = 0.047). A total of nine VLNT donor sites were found in eight patients. The bilateral lower limb lymphedema patient had received two submental lymph node flaps. The VLNT donor sites included five submental, three supraclavicular, and one omentum. The recipient sites for the previous VLNT were distal, all in the medial malleolus region. The median waiting time between VLNT and LVA was 41.4 (22.3–97.9) months. The median volume gained in the lymphedematous limb was 3836 (2505–4584) milliliters (mL) (Table 1). For the bilateral lower limb lymphedema patient, the average contralateral normal limb volume (5528 mL) of three female lymphedema patients of a similar age and height was used as reference value for calculation.

### 3.2. Intraoperative Findings

A total of 72 LVs were found, with a median incision of four (3–5) per patient. The median number and diameter of LVs found were eight (7–9) per patient and 0.6 (0.4–0.7) mm. The median LVA performed was eight (7–9) per patient. The total number of ICG (+) LVs and flow (+) LVs were 57 (79.2%) and 64 (88.9%), respectively. The median diameter of ICG (+) LVs and flow (+) LVs were 0.6 (0.4–0.8) mm and 0.6 (0.5–0.8) mm, respectively. The pathophysiological changes in LVs based on lymphosclerosis classification included 8 (11.1%) s0 LVs, 36 (50.0%) S1 LVs, 286 (36.1%) s2 LVs, and 3 (2.8%) s3 LVs. A total number of 42 recipient veins were found. The median number of the recipient vein found was five (4–6) per patient. The median diameter of recipient veins was 0.8 (0.8–1.0) mm. The median time for an LVA procedure was 455.5 (389.0–510.0) min (Table 2).

### 3.3. Post-LVA Outcomes

**Figure 3:**
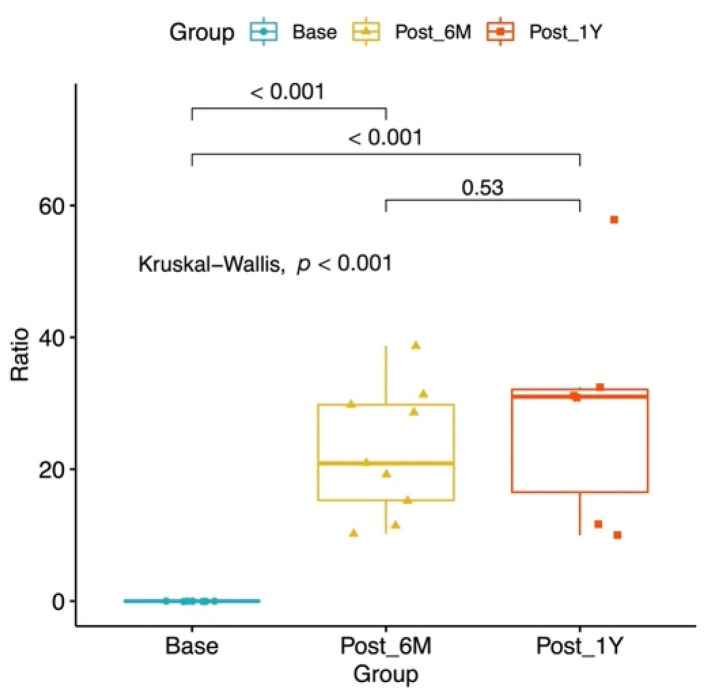
Boxplot for Post-LVA percentage volume reduction.

The median post-LVA follow-up period was 18 (6–30) months. Six months after LVA, the median volume reduction of the lymphedematous limb, in mL and percentage, was 522 (429–1644) mL and 20.9 (15.3–29.8) %, respectively. The 1-year post-LVA volume reduction was 1943 (603–3674) mL and 31.0 (16.5–32.1) %. Significant 6-month and 1-year post-LVA volume reductions were found compared to pre-LVA volume (both *p* < 0.001). However, no significant difference was found between the 6-month and 1-year post-LVA volume reduction (*p* = 0.53) (Table 3, Figure 3).

## 4. Discussion

Instead of LVA, VLNT was recommended as a treatment option for moderate-to-severe lymphedema [22,23,24,25]. However, a few mixed results were reported [18,19], including a case report with exacerbated lymphedema after VLNT [26]. Possible causes of suboptimal post-VLNT results include poor lymphangiogenesis and neo-lymphangiogenesis [2,27]; the quantity of LNs in the LN flap, which was shown to possess direct correlations with lymphatic drainage in animal models [28]; ischemic reperfusion injury during VLNT [29,30,31]; and pedicle complications (arterial or venous occlusion) during or after VLNT [30]. 

What are the alternative treatment options when the post-VLNT outcome is less than ideal or when patients demand greater improvement? Procedures such as liposuction, the Charles procedure, and additional VLNT possess their own advantages and disadvantages. Liposuction can achieve satisfactory results, but persistent and life-long use of a compression garment is inevitable [32]. The use of a compression garment is not well-tolerated in tropical and subtropical climates, such as the study region. Although rare, liposuction-associated fat emboli, hematoma formation, and infections raise concerns [33]. The Charles procedure is usually reserved for severely deformed patients. Skin grafting for a large area with unsightly scars and a long recovery period is not uncommon [34]. Most importantly, these abovementioned procedures do not resolve the issue of obstructed lymphatic return. When additional VLNT is considered, rare but devasting complications, such as flap failure and iatrogenic lymphedema [35,36,37], should be kept in mind. Other issues such as LN flap donor site availability, donor site cosmesis, and the location of the recipient site should also be considered.

In this study, these VLNT patients suffered from lymphedema for a median of 10.5 (4.9–15.3) years, with a median waiting period of 41.4 (22.3–97.9) months before they received LVA as their secondary treatment. The median volume gained in their lymphedematous limbs was 3836 (2505–4584) mL, which is more than what we published in our previous article [6] that noted a median volume gain of 1056.8 (2075.5–2875.3) mL from 90 moderate-to-severe lymphedema patients. The majority of VLNT donor sites were in the submental region (55.6%).

During LVA, a total of 72 LVs were identified. The ratio of lymphosclerosis classification found in these VLNT patients was similar to regular lymphedema patients who had received LVA as their primary treatment in our previous publication [21] (s0: 11.3% vs. 11.3%; s1: 50% vs. 46.9%; s2: 35% vs. 39.3%; and s3: 3.7% vs. 2.4%), signifying that, despite having VLNT for a median of 41.4 months before receiving LVA, the degree of lymphosclerosis of the LVs was not much different from regular lymphedema patients, making LVA a feasible procedure in these patients. The percentage of ICG-enhanced LVs found in this study was higher than in our previous publication [6] (79.2% vs. 70.1%, respectively), but it is also a favorable indication regarding the performance of LVA.

Regarding the post-LVA outcome, these VLNT patients had a significant percentage in volume reduction in their lymphedematous limbs at 6 months and one year after LVA, especially when compared to the pre-LVA volume (both *p* < 0.001). However, no difference was found in the percentage volume reduction between 6-months and 1-year post-LVA (*p* = 0.53). This finding suggests that patients had greater limb volume reduction in the first 6 months following LVA. A possible explanation may be that the pressure buildup in the lymphatic lumen due to lymphedema can result in a higher-pressure gradient in the lymphatic vessel compared to the recipient veins. This pressure gradient can drive lymphatic fluid into the recipient vein, resulting in a relatively fast post-LVA volume reduction in the first 6 months. However, when this pressure gradient is diminished after a lymphedema reduction, the whole process of volume reduction declines. This is the first study to demonstrate the use of LVA on post-VLNT patients. The combined use of VASER-assisted liposuction and LVA was also reported [38].

**Figure 4:**
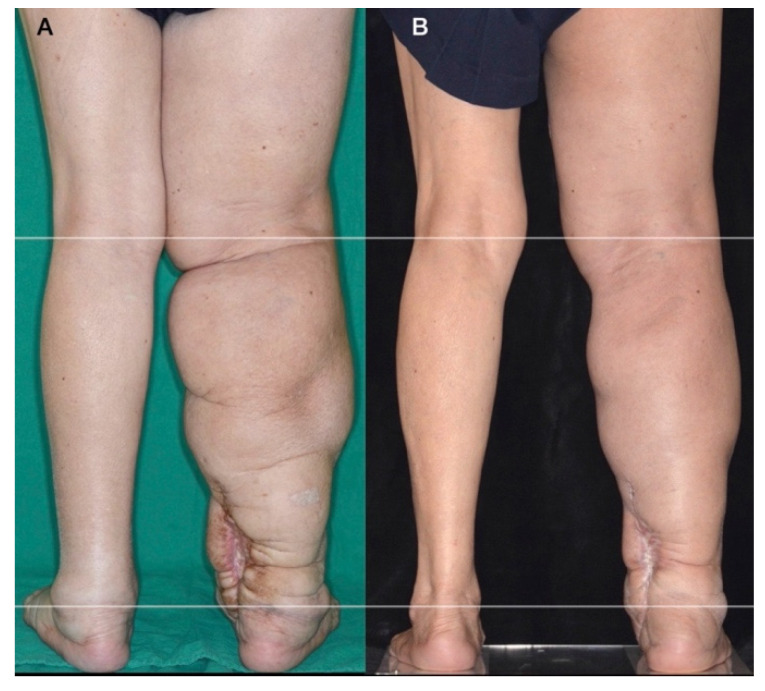
(**A**) A 72-year-old woman with a BMI of 25.8 kg/m^2^ underwent ablative surgery and lymph node dissection 24 years ago due to cervical cancer. No postoperative chemoradiotherapy was performed. She suffered from right lower limb stage III lymphedema 8 years ago with one cellulitis episode. Six years ago, supraclavicular VLNT with a skin graft was performed above her right medial malleolus as a recipient site. Unfortunately, pedicle vessel re-exploration was required the next day, as explained by the patient. Progressive lymphedema with chronic ulceration with occasional lymphatic leakage was noted on the recipient site. (**B**) LVA was performed 3 years after VLNT, with a total of four LVAs performed. Observed is the 2-year post-LVA follow-up.

The limitations of this study include the following: (1) A small number of post-VLNT patients were enrolled. Only eight patients were enrolled in this study. Many VLNT patients did not receive LVA for two main reasons. First, they were reluctant to receive further surgery, and second, they were unaware that their lymphedema could be improved further with LVA. However, supporting articles are currently being published regarding the use of LVA on severe lymphedema [6,7,8]. Formerly, VLNT was the only treatment option recommended for severe lymphedema [5]. (2) No control group was available since we only performed LVA at our institution. (3) Very limited VLNT patient data were available since the patients came from other institutions. These valuable data include pre-VLNT limb volume, the status of LN flap, including the number of LNs, the ischemia time during transfer, or whether any vascular event such as flap pedicle thrombosis had occurred. A patient mentioned her pedicle vessel re-exploration after VLNT (Figure 4). (4) The answer to whether post-VLNT patients who already had good improvements could still benefit from LVA remained uncertain, this is our future endeavor. Based on the results from this study, we suggest that LVA can benefit post-VLNT patients, with limited improvements.

## 5. Conclusions

Among all the treatment options available for lymphedema, such as liposuction, the Charles procedure, or an additional VLNT, the authors recommend the use of a minimally invasive procedure such as LVA as a secondary procedure for post-VLNT patients seeking further improvements. 

## Figures and Tables

**Table 1:** Patient demographics (*n* = 8 with 9 limbs).

Sex, female/male, *n* (%)	7 (87.5)/1 (12.5)
Age, year, median (IQR)	69.5 (58.8–71.3)
Etiology, Gynecologic Cancers */sarcoma, *n* (%)	7 (87.5)/1 (12.5)
ISL Staging (0–I/II–III), *n* (%)	1 (12.5)/7 (87.5)
BMI, kg/m^2^, median (IQR)	26.5 (22.3–33.6)
DM, yes/no, *n* (%)	2 (25)/6 (75)
HTN, yes/no, *n* (%)	3 (37.5)/5 (62.5)
Affected limb (Left/Right, bilateral), *n* (%)	3 (37.5)/4 (50)/1 (12.5)
Chemotherapy, yes/no, *n* (%)	3 (37.5)/5 (62.5)
Radiotherapy, yes/no, *n* (%)	4 (50)/4 (50)
Duration of LE, year, median (IQR)	10.5 (4.9–15.3)
Cellulitis episode before vs. after LVA, *n*, median (IQR)	2 (1–12) vs. 0.00 (0–1.5), *p* = 0.047
Donor site, VLNT, *n* ^£^	Five submental Three supraclavicular One omentum
Recipient sites, VLNT	All located distally, near medial malleolus region
Time between VLNT and LVA, month, median (IQR)	41.4 (22.3–97.9)
Volume gained in the LE Limb ^@^, mL, median (IQR)	3836 (2505–4584)

Non-normally distributed data are expressed as median (inter-quartile range (IQR), 25–75%). LVA: lymphaticovenous anastomosis; VLNT: vascularized lymph node flap transfer; ISL: International Lymphology Society; BMI: body mass index; DM: diabetic mellitus; HTM: hypertension; LE: lymphedema. * Gynecologic cancers included: cervical cancer, endometrial cancer, and ovarian cancer. ^£^ Eight patients with nine VLNT donor sites. One bilateral lower limb lymphedema patient received two submental lymph node flaps. ^@^ Equals preoperative lymphedematous limb volume minus contralateral normal limb volume for nine limbs. For the bilateral lower limb lymphedema patient, the average contralateral normal limb volume (5528 mL) of three female lymphedema patients with similar age and height was used as a reference value for calculation.

**Table 2:** Intraoperative findings during lymphaticovenous anastomosis (9 limbs).

Total LVs found	72
Incisions per patient, median (IQR)	4 (3–5)
LVs found per patient, median (IQR)	8 (7–9)
Diameter of LVs, mm, median (IQR)	0.6 (0.4–0.7)
LVA performed per patient, median (IQR)	8 (7–9)
Total number (percentage) of ICG (+) LVs, *n* (%)	57 (79.2)
Diameter, mm, median (IQR)	0.6 (0.4–0.8)
Total number (percentage) of Flow (+) LVs, *n* (%)	64 (88.9)
Diameter, mm, median (IQR)	0.6 (0.5–0.8)
Lymphosclerosis Classification, *n*, (%)	
s0 s1 s2 s3	8 (11.1) 36 (50.0) 26 (36.1) 2 (2.8)
Total number of recipient veins	42
Recipient Veins per Patient, median (IQR)	5 (4–6)
Diameter, mm, median (IQR)	0.8 (0.8–1.0)
Operative Time (min), LVA, median (IQR)	455.5 (389.0–510.0)

Non-normally distributed data are shown as median (inter-quartile range [IQR], 25–75%). LVs: lymphatic vessels; LVA: lymphaticovenous anastomosis; ICG (+): indocyanine green-positive.

**Table 3:** Post-LVA outcome for Lymphedema patient s/p VLNT (*n* = 8, with 9 limbs).

		Kruskal−Wallis Rank Sum Test	Mann−Whitney Wilcoxon Test
Post-LVA follow-up, month, median (IQR)	18 (6–30)	-	-
Six-Months Post-LVA Volume Reduction *, mL, median (IQR)	522 (429–1644)	H_0_: (pre-LVA) = (6-Months Post-LVA Volume Reduction **, %) = (1-Year Post-LVA Volume Reduction **, %) *p* < 0.001	-
Six-Months Post-LVA Volume Reduction **, %, median (IQR)	20.9 (15.3–29.8)		*p* < 0.001
One-Year Post-LVA Volume Reduction *, mL, median (IQR)	1943 (603–3674)		-
One-Year Post-LVA Volume Reduction **, %, median (IQR)	31.0 (16.5–32.1)		*p* < 0.001

LVA: lymphaticovenous anastomosis; s/p: status post; VLNT: vascularized lymph node transfer; mL: milliliter. Non-normally distributed data are shown as median (inter-quartile range (IQR), 25–75%). * Median post-LVA volume reduction (mL) = preoperative minus postoperative lymphedematous limb volume. ** Median post-LVA volume reduction (%) = (preoperative lymphedematous limb volume (mL) minus postoperative lymphedematous limb volume (mL)) × 100/volume gained in the lymphedema limb (mL)). For the bilateral lower limb lymphedema patient, the average contralateral normal limb volume (5528 mL) of three female lymphedema patients with similar age and height was used as reference value for calculation.

## Data Availability

The data presented in this study are available on request from the corresponding author.
